# Risk factors for HIV infection among adolescents and the youth: a systematic review*

**DOI:** 10.1590/1518-8345.6264.3696

**Published:** 2022-10-03

**Authors:** Pedro Augusto Bossonario, Melisane Regina Lima Ferreira, Rubia Laine de Paula Andrade, Keila Diane Lima de Sousa, Rafaele Oliveira Bonfim, Nanci Michele Saita, Aline Aparecida Monroe

**Affiliations:** 1 Universidade de São Paulo, Escola de Enfermagem de Ribeirão Preto, Centro Colaborador da OPAS/OMS para o Desenvolvimento da Pesquisa em Enfermagem, Ribeirão Preto, SP, Brasil.

**Keywords:** HIV, Risk Factors, Adolescent, Young Adult, Sexual Behavior, Systematic Review, Nursing

## Abstract

**Objective:**

to identify and analyze HIV infection risk factors among adolescents and the youth.

**Method:**

this is a systematic review whose guide question is: what are the risk factors for HIV infection among adolescents and the youth?” In total, five databases and Google Scholar were searched in December 2021 and the found publications between 2012-2022 were filtered without language restriction. Studies were selected by two independent reviewers. The included materials were subjected to methodological quality evaluation and narrative synthesis.

**Results:**

overall, we included seven studies out of the 26,191 retrieved. All studies were conducted in Africa. We found that the female gender, older age, low schooling, Black ethnicity, multiple sexual partners, inconsistent use of condoms, alcohol consumption, and early sexual onset constituted risk factors for HIV infection in adolescents and the youth.

**Conclusion:**

understanding risk factors underscores the provision of health policies and intervention strategies to strengthen the responsiveness of health services and nursing teams’ care to reduce HIV transmission among adolescents and the youth.

## Highlights

(1) Our knowledge synthesis contributes to the care provided in health services.

(2) The main factors for HIV are related to risk behaviors.

(3) Our results point to the purpose of health policies to prevent HIV.

(4) IWe assessed the importance of different social actors and management bodies to cope with HIV.

(5) This study strengthens the role of nursing in preventing HIV transmission.

## Introduction

HIV infection is still considered a health challenge since estimates show 1.5 million new cases, 10.2 million untreated ones (out of 37.7 million), and 680,000 deaths related to acquired immunodeficiency syndrome in 2020 worldwide (AIDS)^1^.

According to global data, 5.1 million adolescents and the youth lived with HIV worldwide in 2019. In the last 10 years, despite the 46% decline in new infections among adolescents and the youth, *per* seven new HIV infections, two were among individuals aged 15 to 24 years^2^. The countries which Global AIDS Monitoring accompanied indicated greater vulnerability of this population to infection in sex workers, gays and other men who have sex with men, injectable drug users, transsexual people, and persons deprived of liberty^2^.

Some factors, such as the unsatisfactory structure of health services and restrictions to its access, contribute to failures in behavioral and biomedical approaches to the adolescents and the youth, reflecting higher infection rates^1^. Moreover, biological, physical, mental, and social factors occurring during their transition to adulthood can also explain this vulnerability^3^ ^-^ ^4^.

Due to these difficulties, nursing plays an important role in healthcare, strategically contributing to control the HIV epidemic among adolescents and the youth by recognizing and understanding how this population socially represents their vulnerabilities and infection risk factors, such as the non-use of condoms, women’s difficulties of taking them with them, issues related to sexual pleasure for male adolescents, alcohol and/or drug use, multiple partners, difficulties in accessing services and sex education in schools, and incipient dialogue with parents and/or family members^5^.

Thus, nursing professionals, together with multidisciplinary teams, should prioritize public policies focused on psycho-emotional and social dimensions and intervene in environments in which these representations circulate since they may enable the interference in the reality of adolescents^5^. Some strategies, when incorporated into the preventive practices nursing performs in communities and appropriate to the needs and risk profile of this population, can positively impact public health and thus reduce HIV transmission and prevalence^6^ ^-^ ^7^.

Considering that knowledge of the adolescents and the youth’s vulnerability to human immunodeficiency virus infection can help identify failures in healthcare services, research have tried to understand the elements which are consistent with this phenomenon and guide the design of intervention strategies to ensure that health and nursing teams prevent the transmission of the virus. Thus, this study aimed to identify and analyze the risk factors for HIV infection among adolescents and the youth.

## Method

### Study design

This is a systematic review based on the recommendations of the Preferred Reporting Items for Systematic Review and Meta-Analysis (PRISMA)^8^ ^)^ and the stages of the “methodological guideline: how to conduct a systematic review and meta-analysis of observational comparative studies of risk factors and prognosis” recommended by the Brazilian Ministry of Health^9^. The protocol of this research was registered on PROSPERO (CRD42021276566) and published in the Research, Society and Development journal^10^.

A systematic review was chosen since it enables us to gather data, evaluate them individually, and publish the evidence found on a given area of interest to collaborate with decision-making and build knowledge on a given question^11^. For this, a research question was defined; eligibility criteria were chosen to screen primary studies; databases were selected; eligible studies were sought; retrieved articles were screened by reading their titles and abstracts; studies selected in the previous stage were fully read; study eligibility was assessed; data were extracted; the methodological quality of the included studies was evaluated, and their results were synthesized^9^.

### Definition of the research question

To elaborate our guiding question: what are the risk factors for HIV infection among adolescents and the youth?”, the acronym PECO^9^ was used, structured as follows: Population (P) corresponding to adolescents and the youth; Exposure (E) to risk factors; Comparator (C): undefined as it would vary according to the factors analyzed in the studies, and Outcome(O) after HIV infection.

### Eligibility criteria

Primary studies that tested the hypothesis of the existence of some risk factor for HIV infection among adolescents and the youth were included. Adolescents and the youth were considered individuals aged between 15 and 24 years^12^ ^-^ ^13^. Thus, studies whose population included adolescents and the youth who were assessed together with people in the other age groups (0 to 14 years and 25 years or more) were excluded. Moreover, we sought to include observational-analytical studies, regardless of the country in which they were conducted.

### Search for eligible studies

The keywords mentioned in the PECO strategy, their synonyms, and their corresponding versions in Portuguese and Spanish were used to identify the controlled vocabulary in the Descriptors of Science and Health (DeCS), Medical Subject Headings (MeSH), and Emtree indices. We should stress that these databases had been previously searched to identify the free vocabulary also used in the literature.

The following databases were searched by two independent researchers in December 2021: Medical Literature Analysis and Retrieval System Online (MEDLINE), Excerpta Medica Database (Embase), SciVerse Scopus (Scopus), Medical Literature Analysis and Retrieval System Online (LILACS), and Web of Science. Google Scholar was used to search for grey literature. Note our use of vocabulary in Portuguese, English, and Spanish to search LILACS, whereas only English vocabulary was used in the other databases.

**Figure 1:**
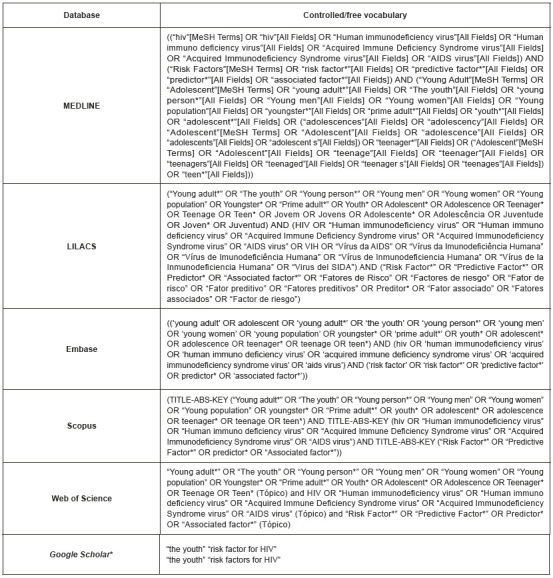
Strategies to retrieve publications used to conduct a systematic review on risk factors for HIV infection among adolescents and the youth, according to database. Ribeirão Preto, SP, Brazil, 2021 *For this platform, two search strategies were used to survey publications belonging to the grey literature

Search strategies were adapted to each database with the use of Boolean operators OR and AND^9^, as shown in Figure 1. In our search, no language limits were used but filters were applied: publication from 2012 to 2022 in all databases and Google Scholar and type of publication (article, conference article, review, systematic review, meta-analysis), except in LILACS and Google Scholar.

### Article screening

The references and abstracts found via database searches were exported to the QCRI Rayyan online systematic review application of the Qatar Computing Research Institute^14^. Then, duplicate publications were excluded before titles and abstracts were read by two independent reviewers. In cases of doubt or disagreement regarding their inclusion, a third reviewer was consulted. To confirm the inclusion of the selected studies, all eligible articles were fully read.

The search process and the eligibility of the materials found and included was shown in a flow diagram, as recommended by the Preferred Reporting Items for Systematic Reviews and Meta-Analyses 2020 Statement (PRISMA)^8^.

### Data extraction

Data were extracted by a pair of reviewers and checked by a third one. For this, a standardized form was used, elaborated according to items suggested by the Joanna Briggs Institute for the extraction of data from systematic reviews of etiology and risk^15^: authors, year of publication, publishing journal, objective, type and setting, population and sample characteristics, recruitment procedures, follow-up or duration, exposure factors (independent variables), dependent variables, data analysis, adjustment for confounding factors, results, and comments. Note that no contact was made with the authors of the articles included to request missing or additional data on any stages of the studies.

### Methodological quality evaluation and synthesis of the included studies

The methodological quality of the included articles was evaluated by a specific instrument to analyze the methodological quality of cross-sectional analytical studies (https://jbi.global/sites/default/files/2021-10/Checklist_for_Analytical_Cross_Sectional_Studies.docx), as recommended by the Joanna Briggs Institute^15^, which enabled us to identify the number of items addressed in the selected studies according to the number of items predicted by the instruments. Finally, the results of the included articles were subjected to a narrative synthesis.

## Results

**Figure 2:**
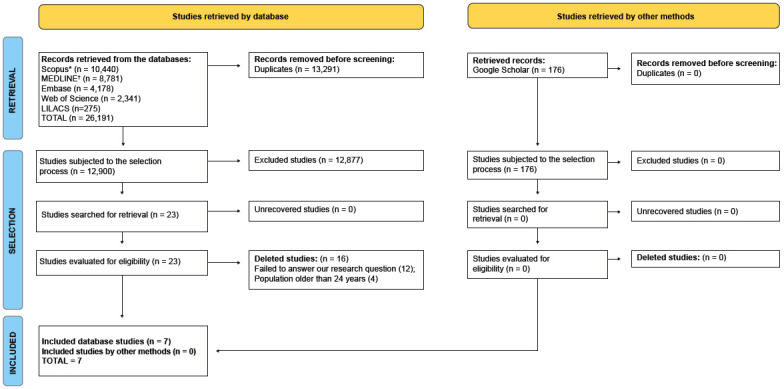
Flowchart of the steps to select articles included in this systematic review on risk factors for HIV infection among adolescents and the youth. Ribeirão Preto, SP, Brazil, 2021 *Scopus = SciVerse Scopus; ^†^MEDLINE = Medical Literature Analysis and Retrieval System Online; Embase = Excerpta Medica Database; LILACS = Latin American and Caribbean Health Sciences Literature

Source: Adapted from Page, et al. (2021)^8^

We retrieved 26,191 studies published between 2012 and 2022 in the chosen databases, excluding 13,291 duplicates. Then, independent researchers read 12,900 titles/abstracts to evaluate their inclusion in this review and 23 underwent full reading. Of the possible eligible studies, we excluded 12 which failed to answer our guiding question and four for failing to cover the population aged from 15 to 24 years. Finally, we included seven articles to extract and compile data (Figure 2).

**Figure 3:**
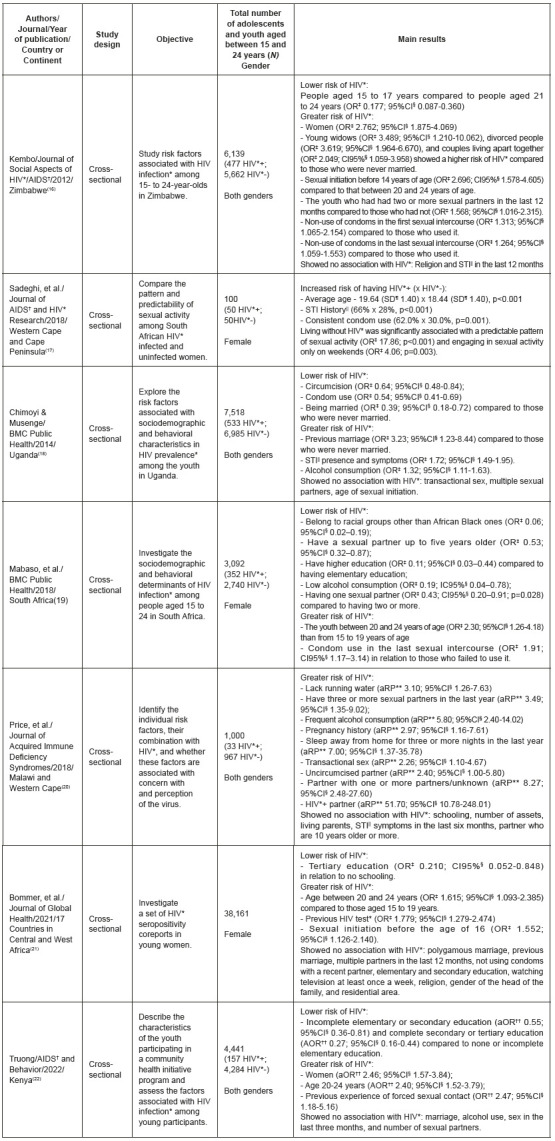
Description of the articles included in this systematic review on risk factors for HIV infection among adolescents and the youth. Ribeirão Preto, SP, Brazil, 2021 *HIV = Human immunodeficiency virus; ^†^AIDS = Acquired human immunodeficiency syndrome; ^‡^OR = Odds ratio; ^§^95%CI = Confidence interval (all were 95%); ^||^STIs = Sexually transmitted infections; ^¶^sd = Standard deviation; **aPR = Adjusted prevalence ratio; ^††^aOR = Adjusted odds ratio

All studies included in this review had been published in English^16^ ^-^ ^22^, in Africa^16^ ^-^ ^22^ in 2012^16^, 2014^18^, 2018^17^ ^,^ ^19^ ^-^ ^20^, 2021^19^ and 2022^22^. Studies showed a cross-sectional design and were conducted in the following countries: Western Cape^17^ ^-^ ^20^, Malawi^20^, Cape Peninsula^17^, Zimbabwe^16^, Uganda^18^, Kenya^22^, and South Africa^19^. A study reported that it was conducted in 17 countries in Central and West Africa^21^. Among the studied population, three included only adolescents and young females^17^ ^,^ ^19^ ^,^ ^21^ and three collected primary data^17^ ^,^ ^20^ ^,^ ^22^ and four secondary ones^16^ ^,^ ^18^ ^-^ ^19^ ^,^ ^21^ (Figure 3).

**Figure 4:**
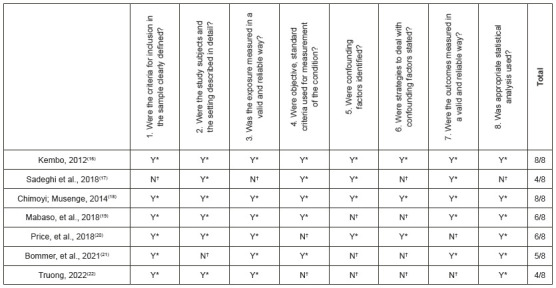
Evaluation of the methodological quality of the articles included in this review. Ribeirão Preto, SP, Brazil, 2021

Source: Questions extracted from Moola, et al., 2020^15^. *Y = Yes ; ^†^N = No

The methodological quality of each publication was analyzed so we could identify their main limitations. In total, two articles contemplated all the items considered for an excellent methodological quality and five failed to achieve such excellence. Moreover, finding and controlling for confounding factors were the elements less addressed in these studies (Figure 4).

## Discussion

All studies included in this review were conducted in Africa, a continent with a high HIV prevalence^23^. AIDS became the main cause of morbidity and mortality in the region due to the influence of the political, economic, and social crises these countries experienced in the 1980s together with the beginning of a global HIV epidemic focused on the historically most vulnerable racial groups. This scenario impacts, to this day, the poorest segments of the population, characterizing pauperization as one of the main social determinants of who falls ill and dies of AIDS^24^.

Regarding the factors associated with HIV in the adolescents and the youth, we found that the older the youth, the higher their risk of infection by the virus^16^ ^-^ ^17^ ^,^ ^19^ ^,^ ^21^ ^-^ ^22^. However, early sexual initiation and absent condom use in their first sexual intercourse contributed to predisposing them to HIV, increasing infection vulnerability^16^ ^,^ ^21^.

Thus, we should mention that early sexual activity indicates the need for health promotion campaigns capable of increasing awareness about individual self-care to prevent HIV and strategies that reach adolescents and the youth not only to inform them but also to build knowledge and collaboration to collectively reduce HIV transmission^21^. We stress the importance of implementing these interventions in an accessible way and in environments capable of providing discussions on health promotion which value sexual and reproductive health (including the prevention of sexually transmitted infections/HIV) among adolescents and the youth even before their sexual initiation.

In total, two studies showed a higher risk of HIV diagnosis among young women^16^ ^,^ ^22^. Studies analyzing gender inequalities in HIV vulnerability in Africa also found this result, showing a feminization of seroprevalence due to aspects related to women’s dependence and submission, difficulties in negotiating condom use, cultural and/or traditional practices, prostitution, and the male refusal to take tests and/or disclose their serological status to partners^25^ ^-^ ^27^ in this population group^17^ ^,^ ^19^ ^,^ ^21^.

Moreover, HIV infection was higher among female groups who perceived themselves outside the infection risk^20^, an aggravating factor of the epidemic which requires actions aimed at reducing behaviors which make people vulnerable to infection. Thus, we stress that the approach of health education actions should align itself with the cultural issues of the African continent and turn to themes such as the consistent use of condoms, reduction of alcohol consumption, and the empowerment of women to negotiate safe sex with their partners^17^ ^,^ ^19^.

We note that African women also show a higher use of health services than men due to gestational, post-gestational, and family planning follow-ups, which leads to a higher testing for STIs/HIV in this population^23^. On the other hand, African testing and counseling services face difficulties to perform HIV serological tests among its young male population^28^ due to, in many cases, cross-cutting issues related to psychosocial, economic, and cultural aspects, such as machismo, perceptions of illness, work, and difficult access to health services^29^.

Research must also understand adolescents/the youth’s relationships with their partners since we found a higher HIV risk among sexual relations with multiple people, partners who have other partners, and who have slept away from their homes for three or more nights^16^ ^,^ ^19^ ^-^ ^20^. It is important to address relationships during health promotion and prevention activities and, whenever possible, guide both partners to testing services, even if having a partner five years older is a protective factor against HIV infections^19^.

Studies must consider other aspects increasing HIV predisposition since we found a higher risk of living with the virus among people who consumed more alcohol^18^ ^-^ ^20^, practiced transactional sex, maintained sexual relations with partners living with HIV^20^, were widowed or divorced, and lived in separate homes^16^ ^,^ ^18^.

We observed such situations especially among women who suffer from scarce resources after the death of their partner or divorce and find a source of income in prostitution. Moreover, studies indicate that divorced the youth tend to consume large amounts of alcohol and have sex without the use of condoms, increasing their vulnerability to HIV^30^ ^-^ ^32^.

The aforementioned gender issues, such as men’s difficult access to health services or low access to serological tests contribute to increasing the transmission of the virus, especially among those who engage in sexual intercourse since timely diagnosis and early treatment tend to be flawed due to partner’ lack of knowledge of their serological status.

Among the factors associated with HIV in women, we found that their partners and other agents forced them into sexual practice, making them more vulnerable to physical and mental trauma and hindering their search for health services due to the stigma of the event. Additionally, health promotion activities are necessary to contribute to reducing the violation of African women’s rights^33^.

Regarding young women who did not live with the virus, we found that they were significantly associated with a predictable pattern of sexual activity and sexual intercourse on weekends^17^, variables which the assessed study should have better elucidated.

Although all chosen studies were conducted in Africa, a continent with a larger number of Black people, studies comparing the ethnicity of the subjects showed that ethnicities other than Black and higher levels of education were a protective factor for HIV^19^ ^,^ ^21^ ^-^ ^22^. In this scenario, post-apartheid socio-historical relations show socially unprotected racial groups living with HIV/AIDS, guided by a residual model of social well-being with income transfer programs aimed at individuals in extreme poverty and inserted in basic social services to the detriment of intersectoral policies and technologies that guarantee access to education and health actions^25^.

This review also found a higher risk of HIV among people with a history of serological testing and STIs^17^ ^-^ ^18^ ^,^ ^20^ ^-^ ^21^. In other words, variables which may indicate the contact of these populations with health services, constituting opportune moments for counseling individuals with sexually transmitted infections and interrupt their chain of transmission, including HIV/AIDS.

We should also mention the importance of receptiveness during counseling since it must contribute to provide rapid STI/HIV testing, ensuring timely diagnoses and the healthcare network instituting treatment and follow-up. In Brazil, the strategy aimed at testing adolescents between 12 and 18 years old takes place without the presence of their legal guardians after evaluating the youth’s physical, psychological, and emotional conditions and its results are provided only to the adolescents/the youth who sought care^34^. Such strategies are important since, in addition to actions of reception and counseling on safe sex, in view of the diagnosis of HIV infection, they can institute antiretroviral therapy (ART) whose biomedical technology contributes to reducing viral load until it becomes undetectable, thus the prospect that the infection will become non-transferable, promoting important ruptures in the virus transmission chain, including in the face of unprotected sexual relations^35^.

Several technologies can reduce HIV transmission but studies still show the need for actions aimed at encouraging condom use since its inconstancy and non-use during sexual intercourse increase the risk of infection^16^ ^-^ ^19^. Hence the importance of expanding the most vulnerable adolescents and the youth’s access to condoms, including the use of female condoms since this is the population most affected by HIV in Africa.

Moreover, the risk of HIV decreases after circumcision^18^ and care and scientific research should highlight it as a protective factor against infection to promote and encourage it. Moreover, policies must stimulate other healthcare technologies, such as Combined Prevention, which consists of: post-exposure prophylaxis (PEP), pre-exposure prophylaxis (PrEP), vertical transmission prevention, immunization against hepatitis B and the human papilloma virus (HPV), diagnosis and treatment of STIs and HIV, distribution of condoms and lubricating gel, and regular testing for STIs and HIV^36^.

In view of the above, we should mention that all the interventions we proposed in our discussion on HIV prevention among adolescent and the youth should be included and considered in the list of activities developed by nursing since it establishes a greater bond with users of health services and occupies an important position in multi- and interdisciplinary teams.

Thus, this study contributes to advance and synthesize scientific knowledge on HIV risk factors among adolescents and the youth, supporting the development of public policies that consider such specificities and aim at expanding the access of these individuals to strategies which actually help to control HIV.

Regarding our evaluation of the methodological quality of the included studies, we should highlight the need to identify confounding factors and the adoption of strategies to deal with them since such actions constituted the main limitations of the assessed studies.

Regarding the limitations of this review, we stress the exclusion of gray literature and the automated search for other publications which could respond and increase or better validate to the found results. Moreover, we emphasize that the articles we included were all conducted in Africa, whose countries show different HIV burdens in adolescents and the youth and very different cultural aspects from the rest of the world, preventing the extrapolation of their results to other realities.

## Conclusion

Among the factors associated with HIV in adolescents and the youth which stood in this review are the female gender, older age, low education, Black ethnicity, alcohol consumption, inconsistent condom use, early sexual intercourse (before the age of 16), and multiple partners.

These data indicate the importance of developing health policies and nursing care practices directed to the adolescents and the youth showing the risk factors found in the studies included in this review to prevent HIV transmission, ensuring an interdisciplinary and intersectoral dialogue between the different points of the healthcare network and among healthcare providers which includes different social actors and management bodies to advance the fight against HIV infection.
